# Transgenerational inheritance or resetting of stress-induced epigenetic modifications: two sides of the same coin

**DOI:** 10.3389/fpls.2015.00699

**Published:** 2015-09-07

**Authors:** Penny J. Tricker

**Affiliations:** Australian Centre for Plant Functional Genomics, School of Agriculture, Food and Wine, University of Adelaide, Urrbrae, SA, Australia

**Keywords:** transgenerational, epigenetic, stress, re-setting, evolution, methylation, transposable elements

## Abstract

The transgenerational inheritance of stress-induced epigenetic modifications is still controversial. Despite several examples of defense “priming” and induced genetic rearrangements, the involvement and persistence of transgenerational epigenetic modifications is not known to be general. Here I argue that non-transmission of epigenetic marks through meiosis may be regarded as an epigenetic modification in itself, and that we should understand the implications for plant evolution in the context of both selection for and selection against transgenerational epigenetic memory. Recent data suggest that both epigenetic inheritance and resetting are mechanistically directed and targeted. Stress-induced epigenetic modifications may buffer against DNA sequence-based evolution to maintain plasticity, or may form part of plasticity’s adaptive potential. To date we have tended to concentrate on the question of whether and for how long epigenetic memory persists. I argue that we should now re-direct our question to investigate the differences between where it persists and where it does not, to understand the higher order evolutionary methods in play and their contribution.

## Introduction

Molinier et al. (2006) demonstrated that stress-induced epigenetic modification could be inherited through several generations in plants, causing considerable excitement. It had long been recognized that such capacity could allow for epigenetic priming of the progeny with important implications for improving crop plants (Mirouze and Paszkowski, 2011; Rodríguez López and Wilkinson, 2015), releasing cryptic variation (Grant-Downton and Dickinson, 2006), for population level adaptation (Richards, 2008) and adaptive evolution (Jablonka and Raz, 2009). Further examples of transgenerational epigenetic effects have been discovered including phenotypic inheritance (Verhoeven and Van Gurp, 2012) and the inheritance of gene expression (Scoville et al., 2011). Although we now have greater mechanistic understanding of transgenerational epigenetic inheritance (e.g., Crevillén et al., 2014; Kuhlmann et al., 2014) there are still few, multi-generational population or species-level studies (Richards, 2006; Bossdorf et al., 2008; Johannes et al., 2008). These few, however, have allowed us to begin to understand the evolutionary importance of stress-induced epigenetic modifications (Rapp and Wendel, 2005; Bräutigam et al., 2013; Kooke et al., 2015).

DNA cytosine methylation is an important epigenetic modification and is demonstrably heritable through mitosis. *Arabidopsis* epigenetic recombinant inbred line (epiRIL) populations that have no DNA sequence variation but epigenetic variation in DNA methylation were created by crossing wild-type “Columbia” with mutants deficient in DNA methylation. The patterns created by recombination in these *Arabidopsis* epiRILs led to a range of stress-tolerance and phenotypes similar to the natural range in accessions within six to nine generations (Roux et al., 2011). Highly heritable epigenetic quantitative trait loci (epiQTL) for flowering time and primary root length were found, associated with the loss of DNA methylation at differentially methylated regions in the founder line (Cortijo et al., 2014) and amenable for artificial selection. These epiRILs also displayed increased plasticity in response to drought, nutrient and salt stresses (Zhang et al., 2013; Kooke et al., 2015) and associated epiQTL were highly heritable, illustrating the stability of epigenetic modification.

## Stress-Induced Priming

Biotic and abiotic stresses trigger epigenetic modifications in the genome. In particular these modifications regulate the “open-ness” of chromatin to suppress or allow gene transcription, transposition of transposable elements (TEs), nucleosome occupancy and recombination. The effect is an altered epigenome that regulates stress response. In some cases the signature of the stress experience remains in the epigenome after relief from the stress, providing a “memory.” If this memory conditions the response to stress during subsequent development, the organism is said to be epigenetically primed. If the memory of the stress experienced by a parent conditions the response of its progeny, this epigenetic priming may be transgenerational. Following Molinier et al.’s (2006) demonstration of heritable epigenetic response to both biotic and abiotic stresses, transgenerational epigenetic priming of plants has been reported in response to pest and pathogen attacks (Luna et al., 2012; Rasmann et al., 2012; Slaughter et al., 2012) and to abiotic stresses including growth in high salt, UVC, heavy metal contamination, increased evaporative demand, heat and oxidative stresses (Boyko et al., 2010; Rahavi et al., 2011; Tricker et al., 2013; Matsunaga et al., 2015). Experimenters typically repeat experiments with clean seed stocks in controlled conditions and yet reproduce the same epigenetic responses, for example the priming of antibacterial defense (Dowen et al., 2012; Yu et al., 2013). These results suggest that epigenetic priming is targeted.

In *Arabidopsis*, priming for antibacterial defense involves active demethylation of TEs that leads to transcriptional activation of defense regulators via hormonal signals (Yu et al., 2013). DNA hyper- and hypo-methylation are observed epigenetic changes in stress response and priming (Boyko et al., 2010; Verhoeven et al., 2010; Tricker et al., 2012), and the recruitment of stress-induced methylation is regulated by small, non-coding RNAs (short-interfering RNAs/siRNAs; Boyko et al., 2010). This RNA-directed DNA methylation (RdDM) may provide DNA sequence specificity to epigenetic modifications via sequence-complementarity of the siRNAs that recruit DNA methylation. However, DNA methylation is reversible and demethylation is also an important response and priming strategy (Yu et al., 2013; Iwasaki and Paszkowski, 2014). Molecular mechanisms that prevent the transgenerational memory of stress have been discovered (Iwasaki and Paszkowski, 2014) and these can be entrained by repeated cycles of stress (Sanchez and Paszkowski, 2014).

The challenge is to understand the dynamics of epigenetic modifications in response to stress and how these interplay with intra- or inter-generational memory to target priming.

## Epigenetic Inheritance of Memory

The regulation of response to plant growth environment is clearly heritable when heritable is defined as passed from the parent to the progeny. One of the best-known examples is the regulation of vernalization requirement in winter annual *Arabidopsis thaliana*. The requirement for vernalizing temperatures to induce flowering is determined in the pathway involving the flowering repressor FLOWERING LOCUS C (FLC), its silencing and the epigenetic maintenance of silencing during warmer temperatures (reviewed in Baulcombe and Dean, 2014). The epigenetic regulation of vernalization in *Arabidopsis* is passed from parent to progeny, i.e., it is an inherited pathway. However, the accumulated epigenetic modifications themselves are reset in each sexual generation, and it is this re-setting that determines the vernalization requirement anew.

The heritable memory of epigenomic regulation is open to selection. In breeding for the epigenetic component of energy efficiency and stress tolerance in *Brassica rapa* (Hauben et al., 2009), the efficiency advantage of the original population and its epigenome component (phenotype, methylome, transcriptome, histone modification) was highly heritable in successive generations undergoing recurrent selection. High and low efficiency selections had distinct profiles of DNA methylation, histone methylation and acetylation different from the parent and from each other. Epigenomic profiles changed during development in opposite directions but were heritable in a cross. This did not indicate that the epigenomic profile had reached reproductive cells because it could not be fixed in the first rounds of selection. The influence of fluctuations in the environment was not explicitly investigated during this experiment, but lines bred for the epigenetic component of energy efficiency were also more drought tolerant.

## Re-Setting the Epigenome

Transcriptional gene silencing is maintained by DNA methylation and histone modifications. These epigenetic modifiers are regulated during gametogenesis and are correlated with the dynamics of chromatin condensation that produce permissive and repressive states of transcriptional activation. At imprinted genes that display parent-of-origin, allele-specific expression, regulation by cytosine methylation and a Polycomb-Repressive Complex determines differential expression through cell divisions with time (reviewed in García-Aguilar and Gillmor, 2015). Methylation is re-programmed in the different nuclei during gametogenesis (Calarco et al., 2012; Jullien et al., 2012). It has been suggested that this re-programming allows the generation of mobile siRNA signals in companion cells that reinforce silencing of TEs in the embryo. In sperm and male germline microspore cells, asymmetric (CHH sequence) methylation is reduced and 24 nucleotide siRNA from imprinted, maternally expressed genes accumulate in sperm cells. CHH methylation is restored after fertilization, during embryogenesis, and the pattern of DNA methylation and silencing is restored at many TEs and epialleles. However, this inheritance of silencing is progressive and incomplete in the male germline cells before fertilization (Calarco et al., 2012). Likewise, the transitions from spore mother cell to megaspore and gametophyte in the female reproductive lineage are also marked by different repressive and permissive histone composition and by chromatin remodeling, suggesting a pre-meiotic epigenetic influence on post-meiotic development (reviewed in Baroux and Autran, 2015). This re-setting during gametogenesis might allow for the removal of epigenetic modifications accumulated in response to stress or growth conditions during development of the parent. Additionally, it provides a window of opportunity to relax epigenetic suppression of transcription and transposition.

## The Genomic Basis of Transgenerational Epigenetic Response to Stress

Natural variation in DNA methylation has been assayed genome-wide in *Arabidopsis* accessions, maize and soybean inbred lines (Vaughn et al., 2007; Eichten et al., 2013; Schmitz et al., 2013a). Along with other repressive chromatin states, DNA methylation is often associated with transposon-rich centromeric regions of the genomes, recently inserted TEs or duplicated regions, and often accompanied by high concentrations of siRNAs that generate RdDM at retrotransposons (Lister et al., 2008). It has been proposed that these epigenetic mechanisms exist primarily as defenses against potentially harmful genomic elements such as TEs (reviewed in Johnson, 2007).

A number of stresses can mobilize TEs (Grandbastien, 1998) and suppression of heat stress-induced retrotransposition of the *ONSEN* element requires the siRNA biogenesis pathway (Ito et al., 2011). Transposons may cycle between active and silenced states and the invasion of a new TE and eventual silencing can establish epiallelism at proximal genes (Marí-Ordóñez et al., 2013). Variation from new insertions may also create new, regulatory inserts responsive to the inducing stress (Ito et al., 2011) or even new, environmentally-responsive genes (reviewed in Oliver et al., 2013). Federoff (2012) has argued eloquently that, in contrast to the view that epigenetic mechanisms exist to suppress TEs, they have evolved and been preserved precisely to allow expansion, duplication and complexity derived from transposition within genomes, whilst repressing illegitimate recombination. Such a scenario requires that the suppression of TE activation by epigenetic means is relaxed or fluctuating. Marí-Ordóñez et al. (2013) found that the epigenetic suppression of the newly invasive retrotransposon *Evadé* (*EVD*) was sequential so that initial, incomplete post-transcriptional silencing shifted to transcriptional silencing over generations once a copy number of 40 was reached. *EVD* bore the seeds of its own destruction; its molecular suppression of post-transcriptional silencing generated RdDM that led to its transcriptional silencing. These findings (and others reviewed in Ito and Kakutani, 2014) are consistent with the idea that TE-activation and epigenetic suppressors act in concert to allow fluctuation and complexity. It can be proposed that the re-setting of epigenetic states at gametogenesis exists to allow this relaxation.

## The Adaptive Potential of Transgenerational Epigenetic Responses

Although the majority of stress-induced chromatin modifications do not persist past gametogenesis (reviewed in Pecinka and Mittelsten Scheid, 2012), others are faithfully re-acquired, albeit limited to one or a few progeny generations not exposed to the same stress (Boyko et al., 2010; Lang-Mladek et al., 2010). Pecinka and Mittelsten Scheid (2012) argued cogently that there is no conclusive evidence yet for the transgenerational epigenetic inheritance of stress-induced memory in plants, and that such evidence would need to document long-lasting changes of more than two generations that significantly influenced the plant’s stress-responsiveness or adaptation. I argue that there is evidence for long-lasting epigenetically-induced change in stress-responsiveness encoded in the genome, but that it is hard to spot.

If the re-setting of stress-induced epigenetic modification at gametogenesis exists to allow encoding of transgenerational memory at new, responsive elements how might we see its signature in the genome? In some known cases, the epigenetic regulation of stress response is fixed in the genome at TE-derived sequences and heritable: Examples include the siRNA-based silencing of the *UBP1b* gene in *Arabidopsis*, the *AltSB* aluminum tolerance locus of sorghum, and the regulation of desiccation tolerance via inducible siRNAs at the *CDT-1* element of *Craterostigma plantagineum* (Magalhaes et al., 2007; Hilbricht et al., 2008; McCue et al., 2012). In addition, the feedback system that generated the *Mu killer* locus in maize may be highly prevalent. Via siRNAs, *Mu killer* heritably silences the *MuDR* transposon (Slotkin et al., 2003). *Mu killer* derives from an inverted duplication of a partially deleted *MuDR* element (Slotkin et al., 2005) and this derivation of the means of epigenetic silencing from the target is common in many genomes (reviewed in Lisch, 2013) and is subject to purifying selection, at least in rice (Hanada et al., 2009). Coupled with evidence that TE insertions increase the number of stress-responsive genes (Naito et al., 2009) these reports suggest that the relaxation of epigenetic suppression of TEs forms part of an evolvable genomic memory, but that this is largely invisible over evolutionary timescales (Lisch, 2013).

Reversible epigenetic regulation may have advantages in fluctuating environments. Burggren (2015) suggested that the immediate “sunsetting” of a stress-induced epigenetic modification once the stress was removed, could allow for bet-hedging against the possible return of the stress. The progeny of one generation exposed to a stress would maintain the epigenetic capacity to respond but not the stressed phenotype. Being poised for fluctuation—that is having an extra layer of regulation ready for release—would benefit survival if the stress was encountered again. Alternatively, epigenetic phenotypic modifications might “wash-out” over generations so that the phenotypic effects would become undetectable or disappear at population scale (Burggren, 2015).

Epigenetic and genetic variation co-evolve (Schmitz et al., 2013b). This needs to happen so that epigenetic plasticity does not completely buffer evolvability and reduce the correlation between fitness and genotype, slowing selection. Klironomos et al. (2013) modeled the effect of selection on epigenetic as well as genetic variation. They showed that early selection of epigenetic variation could allow for the build-up of neutral genetic variation and faster adaptation in comparison with selection via genetic variation alone. This would allow for population survival by epigenetic adaptation following stress, and act as a stepping-stone to increased genetic fitness. Once genetic fitness had increased and been fixed, epigenetic variations would accumulate neutrally.

**FIGURE 1:**
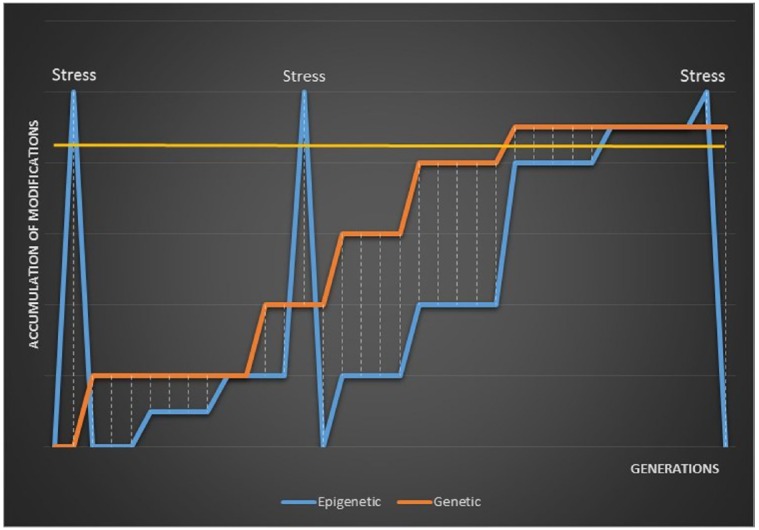
**A model of the distance between epigenetic and genetic variation when epigenetic modifications accumulate following stress and are re-set between generations.** Phenotypic variation is only visible above the gold, horizontal line. The distance between epigenetic modifications and accumulating genetic mutations is illustrated by the dashed lines.

These models might account for the seeming variability in observations of epigenetic phenotypic plasticity and transgenerational epigenetic responses to stress. A rapid accumulation of epigenetic variation in response to stress would be visible in the phenotype of the first one or several generations if washed-out or rapidly sunset. Alternatively, a stress-induced burst of epigenetic modification might be visible in the first generation but largely disappear after re-setting with only a proportion fixed following the re-set generation. A rapid re-setting of stress-induced epigenetic variation followed by neutral accumulation to a new fitness maximum would only become visible once epigenetic variation exceeded genetic variation, or following another stress-induced burst. This model is illustrated in Figure 1.

Theoretically a brief, transgenerational epigenetic memory ensures plasticity, but the dual inheritance of genetic and epigenetic variation ensures adaptation (Pal, 1998). If re-setting allows a window of opportunity for increased epigenetic variation in response to stress, then it could form a vital part of a species’ evolvability. Importantly, mechanistic investigation of re-setting over only a few generations should elucidate the contribution of re-setting to adaptation where, over longer timescales, the evidence of new stress-responsiveness from the activation or exaption of TEs will be invisible. Likewise the recent discovery of molecular mechanisms that restrict transgenerational epigenetic inheritance (Iwasaki and Paszkowski, 2014) will contribute to our understanding of the targeting of epigenetic re-setting or inheritance.

## Conclusion

When we consider the evidence for epigenetic transgenerational inheritance in response to stress we should consider whether it will be visible in an individual epigenome and whether the ebb and flow of visible epigenetic modifications limits or contributes to plasticity. The framework for population and species-level studies of genetics can now be applied in epigenetics to inform our understanding (Richards, 2006; Johnson and Tricker, 2010). It is to be hoped that the combination of mechanistic and theoretical understanding advancing side by side, and the recognition that the persistence or reversibility of transgenerational epigenetic modifications are really two sides of the same coin, will allow us to exploit the undoubted potential of epigenetic regulation of plant stress response for the future.

### Conflict of Interest Statement

The author declares that the research was conducted in the absence of any commercial or financial relationships that could be construed as a potential conflict of interest.
